# Non-Complexed Four Cascade Enzyme Mixture: Simple Purification and Synergetic Co-stabilization

**DOI:** 10.1371/journal.pone.0061500

**Published:** 2013-04-09

**Authors:** Suwan Myung, Y-H Percival Zhang

**Affiliations:** 1 Biological Systems Engineering Department, Virginia Polytechnic Institute and State University (Virginia Tech), Blacksburg, Virginia, United States of America; 2 Institute for Critical Technology and Applied Science (ICTAS), Virginia Polytechnic Institute and State University, Blacksburg, Virginia, United States of America; 3 Cell Free Bioinnovations Inc., Blacksburg, Virginia, United States of America; 4 Gate Fuels Inc., Blacksburg, Virginia, United States of America; Universidad de Granada, Spain

## Abstract

Cell-free biosystems comprised of synthetic enzymatic pathways would be a promising biomanufacturing platform due to several advantages, such as high product yield, fast reaction rate, easy control and access, and so on. However, it was essential to produce (purified) enzymes at low costs and stabilize them for a long time so to decrease biocatalyst costs. We studied the stability of the four recombinant enzyme mixtures, all of which originated from thermophilic microorganisms: triosephosphate isomerase (TIM) from *Thermus thermophiles*, fructose bisphosphate aldolase (ALD) from *Thermotoga maritima*, fructose bisphosphatase (FBP) from *T. maritima*, and phosphoglucose isomerase (PGI) from *Clostridium thermocellum*. It was found that TIM and ALD were very stable at evaluated temperature so that they were purified by heat precipitation followed by gradient ammonia sulfate precipitation. In contrast, PGI was not stable enough for heat treatment. In addition, the stability of a low concentration PGI was enhanced by more than 25 times in the presence of 20 mg/L bovine serum albumin or the other three enzymes. At a practical enzyme loading of 1000 U/L for each enzyme, the half-life time of free PGI was prolong to 433 h in the presence of the other three enzymes, resulting in a great increase in the total turn-over number of PGI to 6.2×10^9^ mole of product per mole of enzyme. This study clearly suggested that the presence of other proteins had a strong synergetic effect on the stabilization of the thermolabile enzyme PGI due to *in vitro* macromolecular crowding effect. Also, this result could be used to explain why not all enzymes isolated from thermophilic microorganisms are stable *in vitro* because of a lack of the macromolecular crowding environment.

## Introduction

Synthetic biology is the engineering-driven construction of increasingly complicated biological entities from simple and basic building blocks to modules to systems. Synthetic biology projects can be divided into two classes: *in vivo* and *in vitro* [1], [2]. *In vitro* synthetic biology is a largely unexplored, compared to living biological entity-based synthetic biology [2]–[5]. Cell-free biosystem for biomanufacturing (CFB2) is the implementation of complicated biochemical reactions by the *in vitro* assembly of a large number of (purified) enzymes and (biomimetic) coenzymes for the purpose of biomanufacturing rather than of fundamental research [2], [5]. CFB2 is an emerging biomanufacturing platform for the production of a variety of products, where CFB2 can do better than microorganisms and chemical catalysts. CFB2 has numerous potential applications, such as the production of hydrogen [6]–[8], of alcohols [9], of organic acids [10], [11], of jet fuel [12], of proteins [13], CO_2_ utilization [14], [15], enzymatic fuel cells [16], [17], and so on.

CFB2 could be economically advantageous over microbial fermentation for the production of biocommodities only when all enzymes in cell-free biosystems have total turn-over number (TTN) values of more than 10^7^–10^8^ mole of product per mole of enzyme and the low-cost bulk enzyme production and purification are available [2], [5]. To obtain high-stability enzymes, the discovery and utilization of thermophilic enzymes from extremophiles could be a shortcut compared to labor-intensive protein engineering and enzyme immobilization [2], [5], [18]. For example, it has been reported that numerous thermoenzymes have TTN values of more than 10^7^ mole of product per mole of enzyme, such as *Clostridium thermocellum* phosphoglucomutase (PGM) [19], *Thermotoga maritima* ribose-5-phosphate isomerase (RpiB) [20], *T. martima* 6-phosphogluconate dehydrogenase (6PGDH) [21], and *T. martima* fructose bisphosphatase (FBP) [22]. To purify recombinant enzymes at low costs, several non-chromatographic scalable methods have been developed, such as heat precipitation [20], [21], [23], ammonium sulfate precipitation, cellulose binding module-based protein purification [24], elastin-based protein purification [25], and so on.

Not all thermophilic enzymes from thermophilic microorganisms are stable *in vitro*. For example, the purified phosphoglucose isomerase (PGI) from *C. thermocellum* was deactivated rapidly at 60°C when its mass concentrations were low [26]. It was speculated that most intracellular enzymes should be stable enough to maintain their basic metabolisms rather than to be repaired or reproduced. This difference in enzyme stability *in vitro* and *in vivo* may be explained by that most intracellular enzymes are more stable due to macromolecular crowding effects [27], [28]. It was interesting to investigate whether macromolecular effects exist or not in cell-free biosystems.

**Figure 1:**
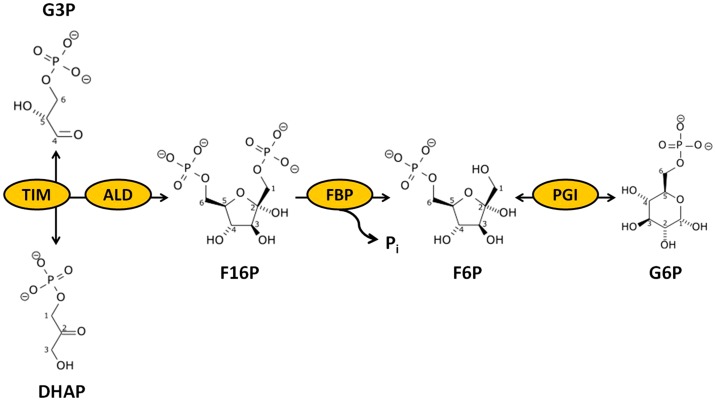
The cascade reactions catalysed by the TIM, ALD, FBP and PGI.



To investigate macromolecular crowding effect on the *in vitro* enzyme mixture, the stability of the four-enzyme mixture containing *Thermus thermophilus* triose phosphate isomerase (TIM), *T. maritima* fructose bisphosphate aldolase (ALD), *T. maritima* FBP and *C. thermocellum* PGI was studied at 60°C. These four TIM, ALD, FBP, and PGI can be regarded as a biocatalytic module in the gluconeogenesis and pentose phosphate pathways (Fig. 1), which was very important for high-yield hydrogen production from sugars [6], [7], [29]. In it, TIM converts reversibly glyceraldehyde-3-phosphate (G3P) to dihydroxyacetone phosphate (DHAP) (Equation 1); ALD catalyzes the reversible aldol condensation of G3P and DHAP to fructose 1,6-bisphosphate (F16P) (Equation 2); FBP catalyses the irreversible conversion of F16P to fructose 6-phosphate (F6P) (Equation 3); and PGI reversibly converts fructose-6-phosphate (F6P) and glucose-6-phosphate (G6P) (Equation 4).

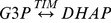
[1]




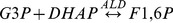
[2]




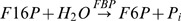
[3]




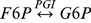
[4]


## Materials and Methods

### Chemicals and strains

All chemicals were regent grade, purchased from Sigma-Aldrich (St. Louis, MO) and Fisher Scientific (Pittsburgh, PA), unless otherwise noted. Avicel PH105, microcrystalline cellulose, was purchased from FMC (Philadelphia, PA). Regenerated amorphous cellulose (RAC) with a high adsorption capacity was made from Avicel [24]. The *T. maritima* genomic DNA was purchased from the American Type Culture Collection (Manassas, VA). *E. coli* BL21 Star (DE3) (Invitrogen, Carlsbad, CA) containing a protein expression plasmid was used for producing the recombinant protein. The Luria-Bertani (LB) medium was used for *E. coli* cell growth and recombinant protein expression supplemented with 100 µg/mL ampicillin or 50 µg/mL kanamycin. The oligonucleotides were synthesized by Integrated DNA Technologies (Coraville, IA). Liquid glucose reagent based on hexokinase/glucose-6-phosphate dehydrogenase was purchased from Pointe Scientific Inc. (Canton, MI).

### Plasmid construction

The plasmids are summarized in Table 1. Plasmid pET20b-tim has an expression cassette containing the *tim* gene [12]. Plasmid pET28a-ald whose expression cassette contains only *ald* gene was kindly provided by Dr. J.J. Zhong [30]. The pCIF plasmid encoding the CBM-intein-FBP fusion protein [22] and pCIP plasmid encoding the CBM-intein-PGI fusion protein [26] were described elsewhere.

**Table 1:** Plasmids and purification methods.

Plasmid	Characteristics	Target protein and purification method	Ref.
pET33b-tim	Kan^R^, *T. thermophilus* triose phosphate isomerase (*Ttc*TIM) expression cassette subcloned into pET33b.	TIM, heat treatment and ammonium sulfate precipitation.	[12]
pET20a-ald	Kan^R^, *T. martima* ALD expression cassette cloned	ALD, heat treatment and ammonium sulfate precipitation	[30]
pCIF	Amp^R^, with cbm-intein-fbp expression cassette cloned (*fbp* gene from *T. martima*)	FBP, bio-specific adsorption of CBM tagged intein-FBP on RAC followed by intein self-cleavage.	[22]
pCIP	Amp^R^, with cbm-intein-pgi expression cassette cloned (*pgi* gene from C. *thermocellum*)	PGI, bio-specific adsorption of CBM tagged intein-FBP on RAC followed by intein self-cleavage.	[26]

### Recombinant protein expression and purification

For the preparation of TIM and ALD, two hundred milliliters of the LB culture containing 50 µg/mL of kanamycin in 1-L Erlenmeyer flasks was incubated with a rotary shaking rate of 250 rpm at 37°C. After the absorbance (A_600_) reached *ca.* 1.2, the recombinant protein expression was induced by adding IPTG (0.1 mM, final concentration). The culture was incubated at 37°C for 4 h. The cells were harvested by centrifugation at 4°C, washed twice by 50 mM of Tris-HCl buffer (pH 7.5), and re-suspended in a 15 mL of 30 mM Tris-HCl buffer (pH 7.5) containing 0.5 M of NaCl and 1 mM of EDTA. The cell pellets were lysed by Fisher Scientific Sonic Dismembrator Model 500 (5-s pulse on and off, total 360 s, at 20% amplitude) in an ice bath. After centrifugation, the target proteins (TIM and ALD) were purified through heat treatment at 60°C for 20 min followed by gradient ammonium sulfate precipitation. The expression and purification of tag-free FBP and PGI were described previously [22], [26].

## Activity Assays

The activity assay of all enzymes was conducted by based on initial reaction velocities. For TIM assay, G3P was the substrate and DHAP was the product. The product DHAP was measured by using glycerol 3-phophate dehydrogenase (GPDH) in the presence of NADH and the consumption of NADH was measured at 340 nm. Because thermophilic glycerol-3-phophate dehydrogenase was not available and NADH was not stable at high temperatures, thermophilic TIM activity was measured by using a discontinuous means. Specifically, the generation of DHAP by using TIM was measured on 2 mM of G3P in 100 mM HEPES buffer (pH 7.5) containing 10 mM MgCl_2_ and 0.5 mM MnCl_2_ at 60°C. The reaction was stopped by addition of 5.8 M HClO_4_ (final, 0.65 M) and keep 5 min in an ice-water bath followed by addition of 5 M KOH until pH ∼7. After centrifugation of the mixture, the supernatants were mixed with 0.2 mM NADH in 50 mM NADH containing GPDH. The consumption of NADH was measured at 340 nm.

The ALD activity was measured by a continuous cascade reaction along with sufficient TIM, FBP, and PGI. G3P and DHAP were substrates and F16P was the product. After the cascade reactions, the reactions were stopped by the addition of HClO_4_ [31]. The final product of G6P was measured by the liquid enzymatic glucose reagent at 37°C for 3 min. The absorbance was read at 340 mM with a reference of the blank ALD solution [31].

FBP and PGI activities were measured as described elsewhere [22], [26].

### Thermostability assays

In the experiments for determining the half-life time of PGI, the residual PGI activities in the absence or presence of the other protein additives (e.g., 20 mg/L of BSA or of the other three enzymes -- TIM: ALD: FBP unit ratio of 5∶1∶1) were measured after the incubation in a 100 mM HEPES buffer (pH 7.5) containing 10 mM MgCl_2_ and 0.5 mM MnCl_2_ at 60°C. The product G6P was measured by the enzymatic glucose kit as described above.

In the experiments for determining the half-life time of the four-enzyme mixture at a concentration of 20, 123 or 617 mg/L, the TIM:ALD:FBP:PGI unit ratio was 5∶1∶1∶1. The residual activities of the four enzyme mixture were measured based on the formation of G6P from G3P (Fig. 1) in a 100 mM HEPES buffer (pH 7.5) containing 10 mM MgCl_2_ and 0.5 mM MnCl_2_ at 60°C.

## Other Assays

Mass concentration of soluble protein was measured by the Bio-Rad modified Bradford protein kit with bovine serum albumin as a standard protein. 12% SDS-PAGE was performed in the Tris–glycine buffer as described elsewhere.

## Results

### Low-cost purification of ALD and TIM

**Figure 2:**
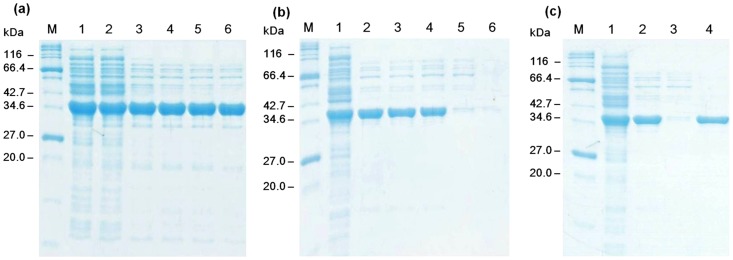
SDS-PAGE analysis of the ALD purification by using heat treatment and ammonium sulfate precipitation. (a) Heat treatment at 60°C. Lane M, marker; lane 1 and 2, the supernatants of the cell lysates induced by IPTG and lactose respectively; lane 3, 4, 5 and 6, the supernatants after heat treatment of the cell lysate for 10, 20, 30, and 60 min, respectively. (b) Ammonium sulfate precipitation. Lane M, marker; lane 1, the supernatant of the cell lysate induced by IPTG; lane 2; the supernatants after heat treatment of the cell lysate for 20 min; lane 3, 4, 5, and 6, precipitated proteins by using 90, 72, 57.6, and 46.1% of saturated ammonium sulfate solution, respectively. (c) Purification of ALD by using heat treatment followed by ammonium sulfate precipitation. M, marker; lane 1, the supernatant of the cell lysate induced by IPTG; lane 2; the supernatant after heat treatment of cell lysate for 20 min at 60°C; lane 3, precipitated proteins from the heat treated sample (lane 2) by using 57.6% of saturated ammonium sulfate solution; and lane 4, the precipitated ALD by using 80% of saturated ammonium sulfate solution from the supernatant treated by 57.6% of saturated ammonium sulfate solution.

One of the obstacles to the economic viability of CFB2 could be high cost of protein purification. Because most *E. coli* cellular proteins deactivated at elevated temperature, recombinant thermophilic proteins expressed in *E. coli* can be purified by heat precipitation. Figure 2A presents the SDS-PAGE analysis for the ALD purification before and after heat treatment at 60°C for 10 to 60 min. *E. coli* BL 21 cells produced a large amount of soluble ALD regardless of the induction of IPTG or lactose (Lane 1 and 2). After heat treatment and centrifugation, most cellular proteins were removed but some minor bands remained. Alternatively, gradient ammonium sulfate precipitation was commonly used as the first step for protein purification. As shown in Figure 2B, ALD in the crude cell lysate was precipitated by using ammonia sulfate more than 72% (Lanes 3 and 4) but not by ammonia sulfate less than 57.6%. A combination of both methods resulted in high-purity ALD: heat pretreatment of ALD-containing cell lysate at 60°C for 20 min; the addition of 57.6% ammonia sulfate, which removed remaining *E. coli* cellular proteins; and the addition of more ammonia sulfate to 80% (final concentration) to precipitate ALD (Fig. 2C, Lane 4).

**Figure 3:**
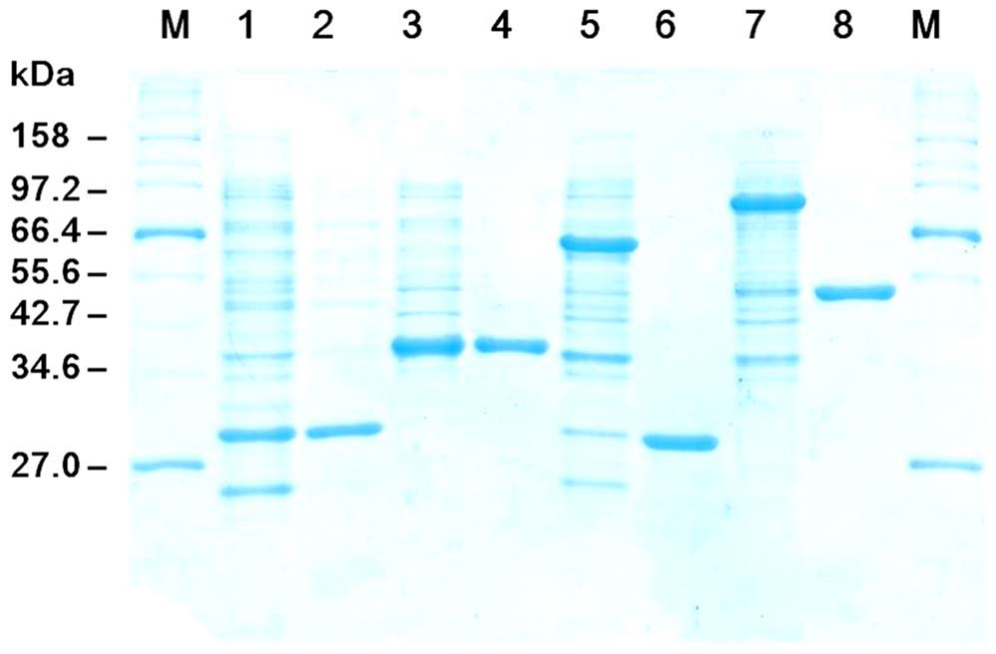
SDS-PAGE analysis of purified TIM, ALD, FBP and PGI. Lane M, marker; lane 1, the soluble fraction of the *E. coli* cell lysate containing TIM; lane 2, purified TIM; lane 3, the soluble fraction of the *E. coli* cell lysate containing; lane 4, purified ALD; lane 5, the soluble fraction of the *E. coli* cell lysate containing FBP; lane 6, purified FBP; lane 7, the soluble fraction of the *E. coli* cell lysate containing PGI; and lane 8, purified PGI.

Similar to ALD, thermophilic TIM was purified by heat precipitation at 60°C for 20 min and 50% saturated ammonium sulfate precipitation that removed other proteins (results not shown). Tag-free FBP and PGI were purified based on affinity adsorption of cellulose-binding-module-containing proteins on cellulose followed by intein self-cleavage, as described previously [22], [26]. All four purified enzymes are shown in Figure 3. The molecular weights of TIM, ALD, FBP and PGI were 28.0, 34.9, 28.6 and 49.3 kDa, respectively, in good agreement with SDS-PAGE results (Fig. 3). The specific activities of TIM, ALD, FBP and PGI were 3500, 1.78, 18.7 and 1900 U/mg protein, respectively. Approximately 125 mg of the purified TIM and 210 mg of the purified ALD were obtained per liter of the culture growing on the LB media, and their respective purification yields were 31.8% and 36.6%.

### Stability of PGI enhanced greatly by other proteins

**Figure 4:**
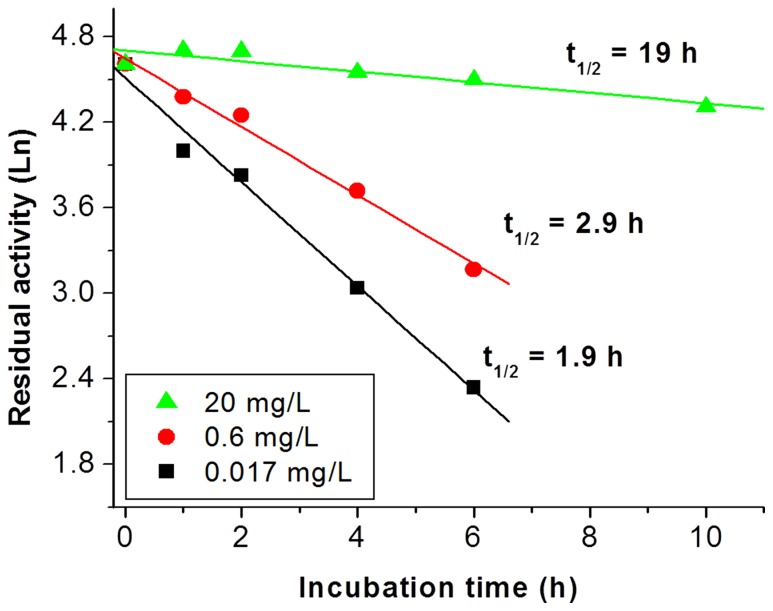
Residual activities of PGI at a different concentration of 0.017 mg/L (▪), 0.6 mg/L (•), or 20 mg/L (▴) at 60°C.

It was found that the thermostablity of PGI strongly depend on its mass concentration (Fig. 4) but not for TIM, ALD, and FBP (data not shown). The half-life time of PGI decreased greatly at 60°C when its mass concentration decreased from 20, 0.6 and 0.017 mg/L. The half-life time of thermo-inactivation was approximately 19 h at 20 mg/L while those of PGI were 2.9 and 1.9 h at 0.6 and 0.017 mg/L, respectively.

**Figure 5:**
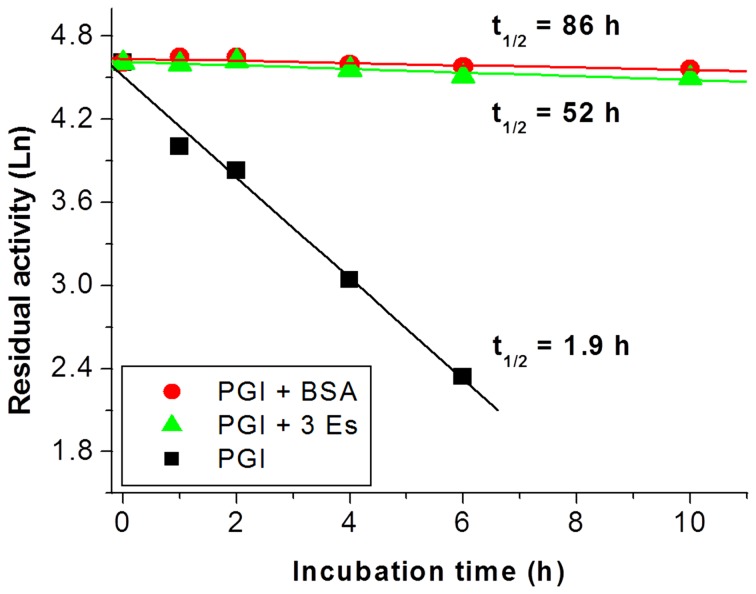
Residual activities of 0.017 mg/L of PGI enhanced by the addition of BSA or the other cascade enzymes at 60°C. PGI only (▪), PGI supplemented with 20 mg/L of BSA (•), and PGI supplemented with 20 mg/L of the other three enzymes: TIM, ALD, and FBP (▴).

It was well-known that the addition of other proteins (e.g., BSA) or other macromolecules could increase the half-life time of unstable proteins. The addition of 20 mg/L BSA into a 0.017 mg/L PGI solution resulted in a great enhancement in half-life time from 1.9 to 86 h by 45-fold (Fig. 5). The addition of 20 mg/L of three enzymes (i.e., TIM, ALD and FBP) also prolonged the half-life time of 0.017 mg/L PGI to 52 h. Both 20 mg/L BSA and three-enzyme mixture stabilized the PGI longer than 20 mg/L PGI only (t_1/2_ = 19 h). This difference in half-life time by the addition of the BSA or other three-enzymes might be caused by different hydrophobic interactions among the surface of the proteins [32], [33]. This result suggested that macromolecular crowding effects might be of importance to keep labile enzymes stable in the cells while low concentration purified enzymes were not stable *in vitro*.

### Prolonged life-time for the optimized four-enzyme cocktail

**Figure 6:**
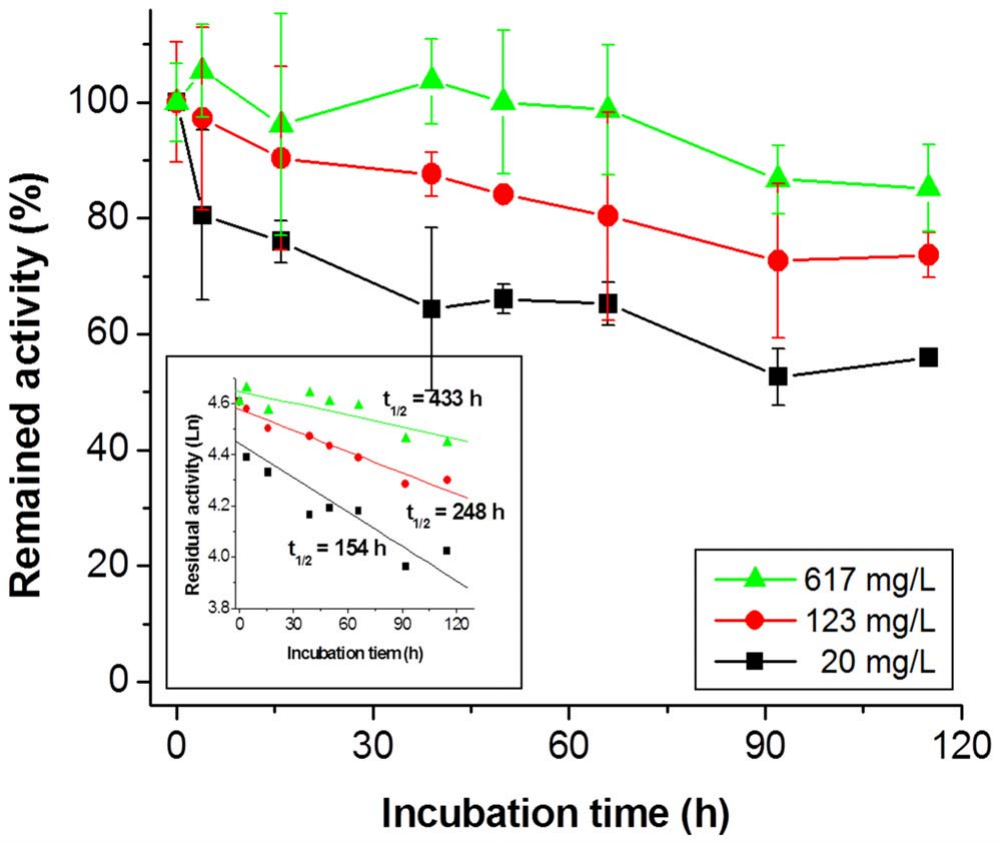
Residual activities of the cascade four-enzyme cocktail that catalyzed G3P to G6P at the overall mass loading of 20 mg/L (▪), 123 mg/L (•), and 617 mg/L (▴) at 60°C.

To further investigate the thermostability of the enzyme mixture that converted G3P to G6P, TIM, ALD, FBP and PGI were mixed at the unit ratio of 5∶1∶1∶1 at their working concentration ranges from 20 to 617 mg/L. The activities of ALD, FBP and PGI were the same, ensuring a constant flux among them at a minimal use of enzyme loading; while TIM activity was five times the others so that there was enough DHAP and G3P for the formation of F16P. The lumped half-life time of the enzyme mixture of 20 mg/L was 154 h (Fig. 6). The lumped half-life times of the four-enzyme mixture were increased 60 and 180% when the mass concentration was increased to 123 and 617 mg/L, respectively. Because of the presence of the other proteins, the half-life time of PGI was 433 h at the total enzyme loading of 617 mg/L (i.e., 1.43, 562, 53.5 and 0.53 mg/L of TIM, ALD, FBP and PGI, respectively), resulting in a great increase in TTN values from ∼2×10^7^ to 6.2×10^9^ mole product per mole of enzyme. These results implied that enzyme stability strongly depends on its environmental macromolecular concentration.

## Discussion

Cell-free biosystems comprised of synthetic enzymatic pathways has numerous industrial benefits: fewer unit operations, less reactor volume and higher volumetric and space-time yield, shorter cycle times and less waste generation, compared to single reactions in cascade [2], [5], [34]. The use of thermophilic enzymes at mesophilic temperatures enables to prolong enzymes' life-time and save enzyme costs greatly. It is relatively easy to over-express recombinant proteins from thermophilic organisms in mesophilic hosts like *E. coli* and purify them by using heat precipitation, such as TIM and ALD (Fig. 2). However, not all thermophilic enzymes (e.g., PGI) were stable enough for heat treatment. Although PGI can be stabilized greatly by enzyme immobilization [26], the presence of insoluble adsorbent decreases efficient reactor volume [35] and could reduce mass transfer on the surface of solid adsorbent [36]. This study clearly suggested that the presence of other proteins had a strong synergetic effect on the stabilization of the thermolabile PGI. Also, this result could be used to explain why not all enzymes originated from thermophilic microorganisms are stable *in vitro* because of a lack of the macromolecular crowding environment [27].

Although it was easy for cell-free biosystems to achieve high product yields [7], [8], [12], it was essential to decrease biocatalyst cost to competitive levels. Enzyme costs are strongly correlated to enzyme product costs and their stability, which was represented by total turn-over number (TTN) (TTN = *k_cat_/k_d_*) [37]. Industrial bulk enzyme production costs have been reduced to approximately $10 per kg, such as cellulase, protease, and so on. It was estimated that enzyme costs in cell-free biosystems would be minimal (e.g., $0.01/kg product) when all enzymes have TTN values of 10^7^–10^8^ [5], [12]. Although free PGI only was not stable enough for meeting the above TTN thresholds, PGI mixed with the other enzymes had TTN value of more than 10^9^ mole of product per mole of PGI, suggesting that there was no further efforts for stabilizing free PGI under their reaction conditions (e.g., 617 mg/L enzyme containing 5000 U/L TIM, 1000 U/L ALD, 1000 U/L FBP and 1000 U/L PGI) [7], [12].

In conclusion, simple low-cost purification method of thermophilic enzymes (i.e., TIM and ALD) was studied by a combination of heat treatment and ammonium sulfate precipitation. The free PGI was not stable while its stability was greatly enhanced in the presence of other enzymes possibly due to *in vitro* macromolecule crowding effects. This synergic stability effect induced by a number of enzymes could be very useful in cell-free biosystems for biomanufacturing.
